# Supplementary material for: DNA Methylation Profile Changes in CpG Islands of Ethylene-Signaling Genes Regulated by Melatonin Were Involved in Alleviating Chilling Injury of Postharvest Tomato Fruit
Source: Int J Mol Sci. 2025 Jun 26;26(13):6170. doi: 10.3390/ijms26136170 (PMC12249976; doi:10.3390/ijms26136170)
Supplement: Supplementary file 1 [file ijms-26-06170-s001.zip › ijms-3713561-supplementary.pdf]

SUPPLEMENTARY S1

ATG in bold indicates the start codon.

Red indicates CpG island.

>SlACS10

-208 AACCAAAAAA AAAATAAAAA AATTTAGGCG GACAAAAAGA TAATAATAAT TCGACCAACT

-148 ATTTGCCACC ATTTTCTCCA AATCTCTTCT CCATCACTCA CCTCCCTTTG TATTCTCCGT

-88 CCACCACCAT CTCGCCGGCC AGGTAGCTGA CTGACGTCAT ACTCCGACAA GGTGACGTCT

-28 TTCTCTACGC CTCCACTATG TCACAGTT**AT G**ACAAGGTCA CGGAACCGTT CACCAACCAG

33 AACAACAACC ATTTCAACTG GCGGCGCCGG CGGCAGAGAC GGAGGAGGAG CCACGACGGC

93 GATGAGGGTG ATAGTACCCT TACAAGGTGT AGTACAAGGT CGGGGTGGTC TTTTCTTAGG

153 TTCAGTAATA CCCTGTGCTC TTTTTTATTT TTGGCAACTC TACCTAAAAC GAAATCGTTC

213 CTCCGGTGGT GACAATAACG GCGAATCTAC GGCACCGGCG AGGTCACCGT CGTCGACCCA

273 TTTGCCAGAA GTGTCTTCTG GGTCTGGGTT ACAAAGGGTT CATTCACGTC TGTTATTGTC

333 ACCGAAGGGA ACTACTGGGC AATCTCAGGT ATCTGCTAGA GCTAATTCGA TTATTTCTAA

393 ACAAATTGAT AGTAGCCCTT ACTATGTTGG ATTGAAAAGA GCTTCTGAAG ACCCTTATGA

453 TGAGTCGAGT AATCCAGATG GTGTTATTCA GCTAGGATTA GCAGAAAACA AGGTAATAAT

513 TTTGAATATT TGGATATATT GAATCATTTT CTAGAGAAAT GTACCAAATG CAATACGTTT

573 CTTGTTGGGA AGTGAATTTT TTTCCTTTCT ACTTTGGTTT GTTGTTTTGA TGATTTTGAA

633 TTTTGTCTCT ATTTGTATGG TTACAGTTGT CACTGGATTT AGTTCAAGAA TGGCTAGCAG

693 AAAATGTGTC AAGATGGATG ATGACTCAAG ATTCGAGTAT AACCGGAATA GCTACTTATC

753 AGCCATTTGA CGGGTTATTG GAGCTGAAAG TGGTAAGCTT TTTAGTTTTT CCTTGCATAC

813 TAAGATGGTT CTTCTTGAAC TATTCAATTA CTAGTAGTTG TTCATATGGT TAATAGTTTT

873 TACAGGCTAG AGAAGGAAAT TGAACAAAAA AATTGCTTGA AAAGGTTTGT AAAAACTTAA

933 AGTTGGTAAT TTGAATCAAG ACTTTATAGG AATAAATCAA TGAGATACCT TTTCAGGTGG

993 TATTAGTTTA TAATTATTAC CTAGGATGGA CTCATGTCAT GTCTATATGC TTGTATAACA

1053 GTTACTCCAT ATCAATTATC ACCCATCCTC TAGTTCTGTG TGCTTTATCG CTGATTTAAT

1113 TTGAAATGTG AATCCCTGGC TGTGGATCGG TAATGGCATT TGACAATCCC AAAGTTACCT

1173 TTCGTATACA AGGGTATTGT ATGTCCCATC TTGAGCTTTC TAAGTGTATA AAGTACAACA

1233 GACATAAGGA AAAGCAATGG TAAGCTTCAC TTTCATTTTA CAGTGCAACT GACAATTAAC

1293 TTCTGAAAAG TTTGTCAACG CTTTAACTTC TACGCTATCG ACGCTAATTA TCATTTTGGA

1353 AAAATAAACA CTGCTCTGAA TGTTTTATAG AAATGATAGG AAAATTTTGA AACCTGAGAA

1413 TGTGTGTGAC CAGACAAAGC ACCAAAACCG TATCCAGAGG GTAATTAAAT TGTTAAACTT

1473 CTCCTACTTG AACATTACAT TGGTAAAACT TGAGTGGAAA CACGTAATTG TTGTGGATCT

1533 AGCTCAGATA TTTATGTTGT TTCAGCTTTT GATAATTGTC TATGGTGGTG TTTGTGCTGA

1593 CCAGAAAAAT AGGACCTTGC AGTGTGATAT GATAAAAAAA TCTAGATTTC GATATTTCAT

1653 CACATTAGAG AATAGTTTGA CCTGCTTTAT TCTGCATGGT TTTTGTTATA AGCAAGTTAA

1713 TAGTGAATAA TGGACTCAGT TGTTCTGAGA GGTGGCCTAT TATTTCCTTG ATTAATACTC

1773 ACCTTGATAC TCATCACTGT GTAACTTATC GAAGGACTAT TGCTAGATTT GCCTGTTGAA

1833 GATGGAAGGC AATTTTGGCC TCTTTGAAAT ATCAGTTGGC TACCTTTGTC ATCAGGTATC

1893 ATATGGGTGG GAGATGATCT GATGGATTTG CCTTTTAAAA TTGCGTGTTT GAGAATTGTA

1953 TGACAGCAAC ATATGAAATT ATCTTTGAGC CACTATGATG ATTGATTCAT AAAAACAATA

2013 AGTGTTTTGT GTGATTCTAC ATAATTATGG AATCTCTTTG AAATGATTTT TATGTTTTCT

2073 CACCTGTATG CAGGCTGTGG GAGAATTTAT GTCTCAAGCT CTAGAGAGAT CAGTGTCCTT

2133 CAGCCCCTCA CAAATGGTTC TTACCGGTGG TGCAACTCCT GCGCTTGAGA TACTGAGCTT

2193 CTGCCTAGCT GATCCTGGAA ATGCTTTTCT TGTTCCCTCA CCATATTATC CTGAGTAAGT

2253 CTCATTCTCC CTCAAAGAAA TTTCGATCTT TTGGTCATTT TTACGTATTT AGTGTATAAA

2313 ATGAGCTCAA TGCATTTGCA GTCTTGATAG GGATGTCAAG TGGAGAACTG GAGTGGAGAT

2373 TATACCTGTT CCTTGCCGCA GTGCAGACAA CTTTAACTTG AGCATTGATG CTCTTGACCG

2433 AGCTTTTAAC CAAGCTAAGA AACGTGGTCT TAAAGTACGA GGGATTATCA TTTCTAACCC

2493 CTCGAATCCT GTGGGCAATA TTTTCTCTAG GGAGACACTT TATAACCTCT TAGACTTTAC

2553 AACTGAGAAG AACATCCATG TAATATCCAA TGAAATCTTG GCAGGGTCAA CTTATGGAAA

2613 TGAAGAATTT GTCAGCATGG CAGAGATAAT TGATTCCGAA GATTTTGACA GGAGCAGGGT

2673 ACACATTGTG TATGGTCTCT CTAAGGACCT TTCTCTTCCT GGGTTCAGGG TGGGGGTCAT

2733 CTATTCTTGC AATGAAAATG TTCTAGCTGC TGCGAAAAAA TTGACAAGAT TCTCATCCAT

2793 TTCAGCCCCA ACACAGCATT TAATCATCCA AATGCTATCA GATGCAAAGT TTGTACAACA

2853 ATTTATCAAA AAGAACAGAG AGAGGCTAAG AAGGATGTCT TCTCTATTTG TCAGTGGATT

2913 GAAGCAGCTG GGAATTGAGT GCACCAGAAG TAGTGGGGGC TTCTATTGTT GGGCTGACAT

2973 GAGCAGGTTA ATTCGGTCGT ATAATGAGAA GGGTGAGATT GAGCTTTGGG ATAATCTACT

3033 AAATGTAGCT AAGATCAACG CAACTCCTGG TTCTTCCTGC CACTGTGTTG AACCTGGGTG

3093 GTTCAGGTTA TGTTTTTCGA CATTAAGTGA GAAGGACATT TCCGCAGTTA TGCAACGTAT

3153 CCAGAAAGTT TTGGAGTTAC GTAAGTCTCT GAGTTAAGTT GCTAATGATA TGATCCCTTA

3213 CATTAGTCAA CCGTGTAATC ATAGGTCCAA AACTTCTGCA GAGATTTTAA GGGTCGAGTG

3273 TTGTGTGATT TTAATGGATC CATAAGTTAT CAGTATCCTG ATAATGAGGT GAGGATGCAG

3333 CAGCTCTGAG AACTAAATGC AGAGAAAACA AAATAAAAAG GCCAAATTGT ATGCAGATGA

3393 TTCCAGAAGT GTGTTCTTGG TCCTCTCCAA CTTTGTCATT TTCACAAATG CATTCCTCCT

3453 CCCAGTTTAT GGATTTTGAA GATAGGTAAA TTAGCAGTCA AAGTTTATGC AAGGAAAAAC

3513 ATATAGCATA TAGAAATGGC TTCAAATGAA ATCCATTTCC AAATTCTTTG TGCATAGTCA

3573 ATAAGTAAGG TCTTAAATCT TGATATTTTC TTATTATTTC TCATTGTTGT ATAGTTACAG

3633 GTAGATGTAT GTACTCATTA TTTGTTCACA TTCATTCTTG TGTTGGCACT AGATATGTAA

3693 TTCGATAAAT GAATCAATTT CTTTCATCCC TT

>LeCTR1

-261 CTTCACGTAT TTACGCACAA AATTCTATGC ATCTTTCAGC CTCATGACCA AACAGCCCCC

-201 ACTTGCTATA ATTAATTCAA GTTAAAGTCC ACAATTCTAC TTTAATAGCT TTTTTACACC

-141 TCAAAATTTC TCAGAATATT ACAAACACAT CCATTTTCTC CATCCTCTTT CTTACTGTGA

-81 GAAAATTTAG AGAGAGAGAA GTGTATTTTG AGCTGAAAAT TGCTAATGGC AGTTGGTAAC

-21 TGAACGTTTT TATACAGGGA A**ATG**TCTGGT AGACGATCGA GTTATACTTT GTTGAATCAA

40 ATTCCTAATG ATAATTTTTT CCAGCCGCCG GCGCCGAAGT TCTCTGCCGG AGCTGGTGTA

60 GCGCCGTACG GTGAGTCTAG TTCTGCTGAG AAGAACAGAG GTAAAGTATT TGATTTGGAC

120 TTGATGGATC AACGCATGAT GCAATCACAT AACCGGGTCG GATCATTTCG GGTACCGGGT

180 TCGATCGGGT CGCAGAGGCA ATCAAGCGAG GGTAGCTTCG GTGGTAGCTC GTTATCTGGG

240 GAGAACTACG TGGGGACTTC CTTTGGGCAT AAGAATGAGG GCTGTGGCTC GTCGGTGGCA

300 AGGAGCTGGG CGCAGCAGAC GGAAGAGAGT TATCAGCTGC AGTTAGCTTT GGCAATAAGG

360 CTCTCTTCAG AAGCAACCTG TGCTGATAGT CCAAACTTCT TGGATCCTGT GACTGATGTA

420 TTGGCATCCC GAGATTCAGA TTCTACTGCA TCAGCGGTAA CAATGTCACA TCGATTGTGG

480 GTATGACATT TATTGCTAGG AATCCTTTGA TAATTTCATA TTGGTAAAAG AGAGAATTAA

540 GAGTAATTGT TTAGTAATGA TTTTCAGCTT TACCAACAGT GAAGGATGTA GTGTAGAAGC

600 TAGTTGGCTC TAACTGGTGT ATACTTTAAA GATTCATTAA ATAAATACAA ATAGTAGACT

660 TTGAACTTAC TCAATCCATA TAGTTTTGAA CTCAAACTCG TAAAGTTCAA TCGTGGATCC

720 ACATATGATT ACAAATGTCT TAGCTGATTA AGTGGTGGAT TGTGGGAGGC TTTGAGTTTC

780 TAATCCAAAA ACTTAAGGTA ATAGGTGAAA TGGTCAACAC CCTTATAATC ACGTAGAACA

840 AGAAATGACT ATATATCATC TCACACATGA ATTACAATAT GGCCCATAAG GGTGTTAGGA

900 TGGGCATTTC AAATGTTGGC TCTGATTTGT CAAAAAATTT GATTATCCAA CCTAAATTCT

960 TTTGACATTA AGTGGAATTA TCCGGGACCT TTTTAAACTC TTTAAGAAGT TCTCAGACAA

1020 CCTATGCAGG ACAACAATAT AACTCAGTTT AGGATTTAGT TTAGACTATT AACGTCTCCA

1080 TGCATAAAGT AGGTGATTAA GCGGGGAAGG AATGTGCTTT TCAAATGCAA ATACATGCAT

1140 AATTATTATA TATTGTAATT GGTGGATCCT TTGTTAGAGT CCTACGTTGA TCAAATATTT

1200 TATATTGAAG TCTCTCTGTA TGGTTTTGGA CAATTTTCAC ATTATAAACT AGCTTTCGGG

1260 GTGAGTTAGA CCCAAAGTAT ATTCATCATG TTACAACTGG ATCCATCCCT TTTTATCATG

1320 AAAAAAGTTC ATTAATTATT CTTGTAGTGT CTTGATTTTG ATTCCTAGCA TCATCTATTG

1380 TTTATTGTGT TTCTGCTATC GCACTATTTT GTTGTTGTTG CTGTTTCCTT ATTAGTTGTT

1440 ATGGTTCTTC ACTGCTTTTC CTTTTCCTAC TTCAATTTGT TGTACTCTAG TTGAGAGTCC

1500 TCCTGAACTC TGCCCCCATG AGATATGAGT ATGATCTGCA TACACTCTAC CCTCCCAAAG

1560 CTCACTTGTG GGATTTCAAT GGGTATGTTG TTGTTGTAAT AATTTTTTAT TAATAATGGA

1620 CAAAAAATAT CAATATACAA GAAGTATACC AAATTGTACA AAAATCTAAA ATATTTGTGA

1680 ACCTCACCTT ATTTTCTAAT GCTTAAGGCT CTCGGCTGAG TAGCAGGAGC AATTTTTAAT

1740 TTATTACTTT ATGACGAGCT CAAACGATGG ATTCAAAATA ATTTCTTGGC AATAACTTTG

1800 TCCACTGAAT AGAAATATTA ATCACATGTT CCATAGTCCT GGGGCTATCA ATTAGCCAAA

1860 CACATGGAAT CCTGCTCTTT CATAAATTCT CAGTTGTAGA GTAACCCAAT GTTGGACCTG

1920 TATAGTAACC CAAAGTTGGA CCTGTAGTCC ACGCTCCAGA GGTCCAACTC TGCTATGTGG

1980 TTGTGTGTTA ATTCCATATT GTTTGAAAGT GGGGATTGAG TTGTGCGTGT CTTAGAGTTC

2040 CAGATGGTTG AGAATATAGA CTGTATTCTC TTTATATGGT TTTATATAAT TCTTAATGCT

2100 TAAACTAGCT CTTGGGATGG AGTTAGGCCT ATTGTTCATT TCTTTAACTT CCTTCCCAAT

2160 TTCAAGAGAT CTTAATAATT CTACTCACAA CGTCTTTATG TTTATACCAT GTGTCAAGAC

2220 TCTTAAATCT CTAAAATTTG ATATGGAGGT GAAAATAATT GATTGCTTGT GACAAGGGAA

2280 GGATGGCCTA GTTGTAATCC ACACCTTCAA CTCTAAGGTT GGGGGTTCAA GTCACTAAAG

2340 GAGCGAAGTG GGAAAGTACA ACTGATGGGC TCTTGCCTCT TGGGTAGGGC TGTAGGAAGT

2400 CAAACTTTTC TAAGTGCTAA TCGCGATCAC CCCTTTTCTT CTTTCTACTT TTCTATTTCT

2460 TCTTTCATTG TTGCCAAATA CAATGAGGCT GAGCTGTTTA CACTAGCTCA AAAGTTTTAA

2520 GCCTTTCTCG TAGTCAAAAT GTGATCTTGG CATTGACAAC CCCCTTCCTT TTTTCTGCAA

2580 GGCCCTAGAA AATGATATGA TAAAAAAAAA GTTTGATCAC ATATTCTTCT GTTAGTTTCA

2640 TGTTTTGCGG TAGGAGCTAC TTCATACTAC CTTGATGTTT TTTTGAATGT AGTTATACTC

2700 ACCTATTGAC TAGATGTTGC CTTTCTATTG TGATTTTAAA TTATCTTACA GATCTCTTCT

2760 CATTCTCAAT TGAGAGTTGA GACAGTCTCT AGATTTCCAG GTCATCATTC CTAATGTCAT

2820 TTTATTGCTC TGGCCTCTTT ATTTATGCCA GATAAATGGA TGTATGTCAT ACTTTGACAA

2880 AGTCCCTGAT GGATTTTATT GGATTTATGG GATGGATCCA TATGTTTGGG CTCTCTGCTC

2940 AGTTGTGCAA GAAAGTGGCC GTATTCCATC AATTGAATCA TTGAGGGCGG TTGATCCCTC

3000 TAAAGCACCA TCTGTTGAAG TGATTTTAAT TGATCGCTGT AATGATCTCA GCTTGAAGGA

3060 ACTGCAGAAT AGAATTCATA GCATATCCCC TAGTTGCATC ACCACAAAAG AAGCTGTTGA

3120 TCAGCTTGCC AAGCTGGTTT GCGATCATAT GGGGTGAGTA CTGAAAGCTT TTTCTTAGAA

3180 GCTCCAACAG ACCCACTTTT TTCCTATTTC TTTTTGTGAT TAGCTTTATT CTCTCTATAA

3240 AGGGGTGCAG CTCCTGCTGG AGAAGAGGAA CTGGTTTCCA TGTCAAAGGG GTGCAGTAAT

3300 GACCTGAAGG ATCGTTTTGG AACTATCGTG CTTCCCATTG GTAGCCTGTC TGTTGGGCTT

3360 TGCAGGCATC GTGCTTTGCT TTTTAAAGTA TGTACTTGTT AAGTTGGTTC TGGTATAAGT

3420 CAGTATATCT AATTTGTAAA GGAAAGATTT AAGGATAAAA TCAGACTAAT GTGTGTATTT

3480 ATCACTAAAT AGAAACCTTG TGGATATTGT ATTTATTTTT TATTTATTCT ACGCTTAGAG

3540 TGATATTGTT TGCAATCCAT AGTGTCTTAG CTTGGTTGTC ATGTACAAGG TTGATGTTTT

3600 ATCATTAACA TCTATGTAAT CATTACAGGT GCTAGCTGAC ATCATTGATT TACCATGTCG

3660 AATTGCCAAG GGATGTAAAT ATTGTAATAG CTCTGATGCT TCCTCATGTC TAGTTCGATT

3720 TGAACATGAC AGGTAAAAGT TCAGATTACA TATGGTGTGT ATTATAATCT TTGGTTCATT

3780 TGAAAGAAAT TTTACTAGGA TATAAAGTGG GAAAGATACT CAATTCACCT TTTGTATACC

3840 TTTTTCCTAG ATAGAATAGT TTTGGGGATG GTGTGTTGAG TCCTTAAACG AATGGTAGTT

3900 GATGCTGCCA CTTACTTTTA TTTGGATAAC TGATACCCCT CATATATATT GTATGAAATG

3960 ATCAAGATTT ACTTTCCTAC TGCTATAATG TGTCTGCTGG TAACATTGTA ATTTGTATAG

4020 ATGCTTTCTG CCTTCTTTCA CGTGGTTATT TTATTCATGC TATGCCCAAC ATCCAATTTT

4080 TCTTCGGTTT CATAAAGCTG GTGAAGAAAA ACAAATTCAA AAAATGATTT ATTTGTCAAA

4140 AGAGCTGCTC AATATATATT CGGAACATGA CTGGCAAAAT TTCTCGTTGT AATAGCTAAT

4200 AATGTGACCT TCCTGATGTG ATAATATATA TCTTTAAAGA TTCAGTCGCT AGCAGAAGTT

4260 TGCTTCTCTT AGTACTTGCT GCAGCATTCT TAGTAATTTA GAATTAAGAT ATTTGAGTGG

4320 AATGGTAGTT CTTACTAATA TGCAGAGATT GAATTTTTTA AATGAAACTT GAAAAAACGG

4380 ATCCACTGTT GATGAGATAG GGAATGTACC TAAAGTTGTC ATACGGTCTT AATCACTCAG

4440 ATTTTAAATC ATGATCCAAA TTCCAATGCA AAAAAATCCT TCTCTTCTTC CATGCAGCCC

4500 CAAATGCAGA TAAGAAGAAC CAAAGTACTA GAAGGAAGTA GAATCAGATA TCCAAAGCAG

4560 ACTGCTACTT TAAGTTGGAA ATAGATTAGT AACTGGATAT GTCTGTTATT TTCATCTGTT

4640 GAGCTTTTCT GCTGGATTCA GTTTACATGA TAAGACATAT ATAGTTTTAA AATATGCTAA

4700 ACATTTGTGT GCTACCTGGT TAGCCTCATA TTAGGAAGGA GTGCTAGTAT TGACATGTAA

4760 CTCTTGACCA TACAGGGAGT ACTTGGTTGA TTTGATCGGT AAGCCTGGAG TTTTAAGCGA

4820 ACCAGATTCC TTGTTGAATG GTCCATCTTC CATCTCAATC CCTTCACCTT TGCGCTTTCC

4880 GAGATACAGA CAAGTTGAGC CTACAACTGA TTTCAGGTCA TTGGCCAAAC AGTATTTCTT

4940 GGATAGTCAA TCACTTAATC TACTGTTTGA TGATTCTTCA GCTGGTGATT ATCTAACTCT

5000 TTAGCTGCTT AAAAAATGCA TTGTATTTTG TTTGTTTTGA ATTGTCCTTT CCCAACATCT

5060 TACTTGGCCC TTGCCATGAT ATGTTGACAA ATATGCAGGT TCATACACAA ATTTAGAATT

5120 GCTTCAGTCT AGCATGCTGC ATTCCACCAA AATGCCCCCT TTTCCCAAGT TCCCTTTCAT

5180 ATTGTGTCAA AATTGTGGTG TCTGATAATC AGTTGAACTT ATTAGAATCT CCTGAAAAGT

5240 TCGCCAGTAT GTGTGGTTAT TTGTCTTACT TGAACGGAGT ATACTTTCAG CTTGCTTATA

5300 ACATTGTTCA ACACTGTAAT GTCTTAAAAC ACAATTGAGT ACATTGACGA TCCAGAGTTT

5360 CAAGTTTTTT TTTGGAACAG GTGAAGTGGT GTATATTATA GAATAAAATT AGAAACCTGG

5420 TGTTAAGCTG GTTCCTTGAT AAGTACAAAG TGTGAAACCC TTCTGTACAA AAACAAAAAG

5480 AAGGTTCTCT CACCAGACTA AGCATCTATT CCTGTAGTGA ATCTATGAAC TCTAACGTAC

5540 CATGTTCATC TTTAACATAT TGTAAGGTAC ACCTCGAGCT AGTCCAGATA CCCCATAATC

5600 GTGCATCCAC ATGAGAATGC AATAACAGGT GGTCCACATT TTCTCTTGAA CATCTGCACA

5660 TAAAGCACTA GCTAACATAT GTGATCCTCT TTTTCCTCAA GTTTCCAGTG GTCAGAACTG

5720 CTCCCTTGCT GCCAACCAAG CCAAGCACAC TTTTTAGGTG TGCTAAGAAG CCAAATAATA

5780 CATGCAGGAA GCCCATCTTT CCTCACTAAA AGCTTGTGGT AGTATGATTT TAATTGGAAA

5840 ACCTCCATCT ATCCTTGCTT CCCACCTCCA CAAGTCCTAT CTACTGAGTG TTGAGTTTTG

5900 TTTCTAGAAA ATTTTGATCA ACCTTTGGAA ATTCATCAGT ATATATACAT GTATATATAC

5960 ATACTTCGCA TCTTCCTTCT TTTTTTCTGG GACAACCTAA ATTTATAATA TGCAAAGGCA

6020 AATTAATAAC ACTAGTGATC TACCTTGTGC CTTGACTTTT ATCTAGGAAA GTATGTAAAT

6080 AAAGTATGAA ATCTATTTTT TTTTAATTGT GCAAATAAAT ATTAGTACTA TTTTGTTTTG

6140 GACGATTTCT CTTCAGTTTT TTTAGTGAAT ACTAGATCCA TCCAGTCCTG TTTCAGAGAT

6200 TCATAATGAT TAGTATTTGA TGTAGGAGCT GCAGCTGATG GAGATGCAGG ACAATCAGAC

6260 AGAAGTTGCA TTGATAGAAA CAATGTAGTC TCTAGTTCAA GTAATCGTGA TGAAATTTCT

6320 CAGTTACCTC TGCCTCCATT AAATGCATGG AAAAAGGGAC GAGATAAAGA ATCTCAACTT

6380 TCTAAAATGT ATAATCCTCG AAGTATGTTA AACCCAGTGA ACATGGACGA GGACCAGGTT

6440 CTTGTGAAGC ATGTTCCTCC ATTCCGGGAA GATGCTCAAT CACCGATGAC ACGACCAGAT

6500 ACAGTAAATG ATACTAGGTT TCTTGCTGGA GGAGGTCATG TTGTTTCTGC TATACCAAGT

6560 GAAGAACTTG ATCTCGATGT AGAAGAGTTC AATATTCCAT GGAATGATCT GGTTCTAATG

6620 GAGAAAATTG GGGCAGGTAA TCTGTCTCCT GGATTTTAGC CATTTGAATA GTGAGCTGTG

6680 GTTGCTTGGT TAAATGTCAG TTATTTTCTG TTATTTTTTA CCCCTTGAGG GGACACATTA

6740 TATGGGATTG TATAGTACCA TAACATTCTT ACTTGATTAA GCTAACATAG TTTGTCTATT

6800 TTCAGGGTCT TTTGGTACTG TTCACCGTGG TGATTGGCAT GGCTCTGTAA GATACTTATC

6860 CAATTAGATC TCAGTTTTTG GAAATCTTCT ATCTGAAAAA ATATCTCAAC GTATCTTCTA

6920 CTTAAGAGAT CCATTTTTGC AATGTGAATT TTTTCATCTC TATTGTACTT CTTTGGGGGG

6980 AAACCGTATT TTTGTTGTAG TTTAATCGAG TTAGCTCTGA AGCTCCCTCT CAAATACTTT

7040 TTACTGGCGT TGTTGTTTCT TTTTCTCTTT CAGATTTTCA CTTTTTTATG TTGGGGAGGA

7100 GGGGTAGTCT ATTTTATGTC TAGTGGAGAT ACAACCATGT GAAGCCTGCT ATATTACGTT

7160 GGTCTTATTC CTTCCTGATT GTATCTCATG GAAGTTTGTA TCATTTAGGA TGTTGCCGTG

7220 AAGATCCTCA TGGAACAAGA TTTTCATGCA GAGCGACTCA AGGAATTTTT GAGGGAGGTA

7280 AGTCTGTGCT TTCTCCCCCA ACATCCGACT AGCAATCAGG AAGAACTAGA AAGGGACCAG

7340 AATGCACGGA GATTGAGGAT CTGCGGACGT CAGTTTGTTA TTTGTATTCC TTCTACATCC

7400 TTCTAATTCT GTTATTTTTT TGGGTGTCCT AGGTTGCAAT TATGAAGCGG TTGCGACATC

7460 CAAATATTGT ACTTTTTATG GGTGCTGTCA TTCAGCCACC AAATTTGTCC ATAGTCACGG

7520 AATATTTATC GAGGTCTTAG CCTATTTCTC TCTTCAGCTT ATTAGAGAAA GCACTTTGTT

7580 TTTCCTGATT TAGCTGACTG GTTGCCATGT TATCATGTAT TTCAGAGGTA GCTTATATAG

7640 ACTTCTTCAT AAACCTGGTG CGAGAGAGGT GTTGGATGAA AGGCGTCGCC TGTGTATGGC

7700 TTACGATGTG GTATGATAAA TGTGTCCTAC TCCTCTCTTC TTTCTCATTT TACTTATTCA

7760 CTGGGGCTGA TCGCTTAATA TGATACCATG CAGGCAAATG GGATGAATTA TCTTCACAAA

7820 CGCAATCCTC CCATTGTGCA CCGAGATTTA AAATCTCCAA ATCTTCTAGT AGACAAAAAA

7880 TATACAGTGA AGGTGAGAGT AAGAACTTTA GCAAGCATAA AGGTTCAATT TTCTTTGTCA

7940 TGATTGATGT ATTCTCAGAC TTGGCTGTCA GAGTAGTCAA ATCTGAAGGA AGCATCATCC

8000 TGGATAGGAC ATTTTTTTTT CCATTTGAGA GGCGTCTTCA AACCCAACTT CCTCAGACAT

8060 TTTCCCAAGG GTCAAGAGAA GCATATAAAA GTTGTAGTTT CAGAATCTTT ATAACCTAAT

8120 CAAAATTGCT GAATAACATA AAGCAAGTAT TTATCAGTTT CTCTGATGAA AGTTAACTTT

8180 TCATTCCATG AAATGAGTTA TTTTTCGATA AGAGAGGATA TTTCTTGAAA ATTCTGTGAA

8240 AAGGGGAAAA ATAATTATAC ATTATTTGAC ATTTTGGAGG AAGTCTGGTC CATAATCTTT

8300 CTTTGTGATC TTCTAGATAT ATGCCATCTA TAAAGTGCTT ATTTCAACTG CCATTTATCC

8360 AGCATGTTTC AAGAAATTTA GGAGTCAATA TGAGAAGGAA GTTTCTATTT CCGGTTTAGA

8420 TTGTCTTTCA AATTTTCTTC TTATGTTTTT CCATGACATA TCTTCTCCTC TTTTTTCAAC

8480 CTCTTGGTGC ATTAATTTTC TTTTTAACAG GAAATATTTA GGGTAGATGT GTCTCCTCTT

8540 ACTCTGGAAA TGCGTGTGTG ATCCTCTTCC TTTTAATCAA CTTGGACCAT CGAGGGAAAA

8600 CTAATAAATT CTTGATCATT CAATTTGACT TTTCAAACCC AACCAAAAAA TGATTTTTGT

8660 AAAAAAATAA GTTCTTGAGA TCTGATTGAG AATTTGCTTG AGAAAGAGGA GGGGTTTTGG

8720 GATGTCCGTG ATTTAAGAAG GCTCTGTCCT TTACAGCTAG CAGCATCCAT AGTGTAGTGT

8780 GACTATGTCT AATATTACCT CTACTTTTAT TTGTGATTGA TGCTTAATGA TGAGAGGAAT

8840 ATTGCAAGTT AACAAATAAT TTACGCTAGA GTTTGTCTAG AATATTGAAA ATAATATATG

8900 AAGCTGTTCT TTCTCTTTGT TCAGTAAAAG GTACTAAATG TTTTCTCTTG TCTAATGTCT

8960 TCATCAGATC TGTGATTTTG GTCTTTCTCG TTTCAAAGCG AATACATTCC TTTCATCAAA

9020 GACTGCTGCC GGAACTGTAA ATTCTTAATT CGACTCTGTT GCCTTTATGT TCTGTTGATC

9080 TTTGACTTTT TTCTTGATGA TTGAATTGGG GAAATAAATG ATTTGGTGCT TATTGTGACA

9140 GCCGGAATGG ATGGCGCCTG AAGTTATTCG TGATGAACCA TCAAATGAGA AATCTGATGT

9200 ATACAGCTTT GGTGTCATTT TGTGGGAGCT GGCAACTCTT CAACAACCAT GGAATAAATT

9260 GAACCCACCA CAGGTTTGTA TTTGTTTGTT TGAAGTATTT TAATGTTAAG AGCATTTAGG

9320 TAAAAATGAT GATTTAAACA GTGCAATCTA GAGGTGAATA GTTTATATAT CAACAAAGGA

9380 CCAAAAGCTA TTTCTTGGGA TGTGAAAATG AGTCGGTCTC ATATGGCTCT GCCAGGGATT

9440 AGGGCACCAC AAGTCGGGAT GAGCCATTCC AGGAAGAGTC TTTTCAATTT GCTCTGCGTC

9500 ATCTACACTC TTCTTCATGT AGTTAATGTA GAAAGCTAGG CATTCATGAG CCATTGAATG

9560 GAAGAGTTCC AGGGGTACTA ATTTCTATTT GTGTGTAGGT TTTATTTTAC TTCTTGTGTA

9620 GTCTTTTAAT AGTTCATATT TTGTGCAACA TATATGTAAA CAGTTTGTCA AAATAATATT

9680 TAAGAAGAGA ACTATGGAAG TGGCGGCGGT GAGGTTGACG GCCAGCATAA AAGTAGTACG

9740 AGTATGATGA ATCATCTTAC TATAATTGTA ATGCCTGCCT ATCCAGACAT GTGCTATATG

9800 AGCACTGGAG TATGTGTTAA ATGAGCTCGG GGTTTCATCA CATCTCCCTC TTAGGTGAGA

9860 GCAAATATTT TTTATTAAAA AGAAAGTGAG AGCAAATATA AGCAAGGTTT CCCACACCAT

9920 ATACAGGTGT GACTAAAGAA ATTAGTCTGA CCACATTGAC GAATTAATGT GGTGCTCTAT

9980 AGGACAAGCT TGCCGTGTGT TAGAGGATGG GGAAGAAGAA GCTAATACAA GAGAGTAAGA

10040 TTCAAATAAG TTGTTGGAAT AGACTTGTTT GACGGGAAAG CAAATAGATT CAGCTCTGCT

10100 AGCATGTGGT GGAGTATCTG GGAAGGAGTG GAATGGAAGA TGTTAGGGGC ATTAGAGCTC

10160 AAGTTCAAAG TTTAATCTGA CTGTATTTTA TGGTCTCTTC CTGGTGTACA ATGGATGTAG

10220 TTGAGGATGC TGAAACCCTG TTACTGTTCA TAGATATTTT GTATTGATGG CCAGGGTTCA

10280 CTTTTTTTTT TCTCTGAGTG TAATCACAAC ACTTTCTTAG TGCTAGTGAC ATCAGTAATA

10340 GTTGCCAGTT CCCAAAAGAG AAGAAGGTGG ATGCTGAAAT CTAGAGATAT TGGTGTTAAA

10400 GAATGACAAA AAATCTCACA TCAGTGGTTA ATGAGATGGG TGGACTCCTT ATAAGGCTTG

10460 GACAATCTTC CTCCCTTGGA GCTAGCTTAT GGGGTGTGAG TTAGGCCTAA GACCTAATTT

10520 CACATGGTAT CAAAGCAGGT GGGGTGTTAA AGAATGACAA AAAGTCGGTT AATGAGATGG

10580 GTGGACTCCT TATAAGGCTT GGCAATCCTC CTCCTTTTGA GTTAGATTTT GGGGTGTGAG

10640 TTAGGTGTAA GACCTAATTT AACAATTGGC AACATATGTT GTAAAATGTG GTATTGTGGG

10700 ATAAAACTGG GAAGAAAGTG GGACTCATTG CAGTATTAGG ATTTAAAGTG AATTGTCGAA

10760 GTAAAGAAAA TCAGCGATAT GATTTATCTC ATTAAGTCTA TTGTAGGAAA AGTATGAACT

10820 CAACATCATG GGTTTCTATG TACTACAAAT AATAGACAAG AGAACTAAAA CAAAAATTTG

10880 GGAAGATATG GATAGTAGGA TAACCTTGGT CGAAGAGATC CCATGGGACT AGAGGCCATA

10940 GAAGGAAGGT AAATGGGAGT TGGAAGCTGA TGTAGATATT GTGGGAAGTA AATGACAAGT

11000 TGCATTATGA AAGCAATAAG AGTAGTCTTT GGTACATTTT AGAGAGGCTT AGGACTTGGG

11060 AGCTCAATTA GAGAAGCGTG TAAAGAGCTG TTAAAATAAA AAGACAGAAC AAATGGATTA

11120 TGAATATCAA AAGCCTTTAA AGAGTATTAT CAATATAAGA AGTCAGTTCC CTCATGTTCT

11180 TGTAGACCTC CATGTGCACC AGTCTATAGG TGCAGCAATA TGCAGGTTGA AGGCGATAAG

11240 AGGGCACAAG CACAAGAAGA AAAGTAGTTT CAGAAGATCA ATACAAGCTT ATGTAAGAAT

11300 AAACATAATA GAAGAAAAAA GATCCATATA GGGTACATTA GCTAGTTGGA TTAACTGTTG

11360 AGTTTAGCTT TTACACTTAT TTTGTGTCCA ATTTTGTGAG AAGTTCTCTT AATTGGGTAG

11420 AGACTCCTAT GTTCATGTAG AAATAAGTTT GATTCATGGA AGACCAAATG AGACAATACA

11480 ATATCAATGC CTCATGATTA TTAAAACTGT TTCTCGTTGA TATATAGTAC ATTCTAGCTT

11540 CTGCTACAAG TTTGATTTTT GAAAGATCAA ATAGAGGATC ATCCGTCAAC CCTCGTGATT

11600 ATTGAACTAT TCCCTTCTCT TTTCTTTTCT TTTCTTTTTT ATTATTGTGA ATGGCAGGAA

11660 ATGTGCTTTA ATGAATTTGA CTTTGTAGGT TATAGCAGCT GTCGGCTTTA ACAGGAAGAG

11720 GCTTGATATT CCAAGTGACT TGAATCCTCA AGTGGCGATT ATTATTGAGG CTTGCTGGGC

11780 TAAGTGTGTT TCTTTTACTT GTTCCCAAGT TTGCACCATT TAGCATCATG ATTGTTTTAA

11840 TGTGTTATCT TAAAGTTACT TTTGATGATG TTGCAGTGAG CCGTGGAAAC GCCCCTCCTT

11900 TTCCACTATC ATGGATATGC TGAGACCTCA TCTTAAATCT CCTCTACCTC CACCAGGTCA

11960 CACAGACATG CAGTTGCTCT CATGAATACC TGTGCTCTCT GCACATATTC TGCCGTCATT

12020 GCAAGCCATG TTTCTGGGAG GAAGAACATT TGGATTATGT CAGGTTTGTC TATCACCCAA

12080 ACTGTTTCTT CGTTACACTG AATTTTAAAG TTATTACCTT GGGTATGTCA CATCTTAGTT

12140 CAATTCTAGG TAAGTTAACT CTAGGTGACT GGTGCCAGCA GTAGTTGAAT AATTTGGTAG

12200 AATATTTGGA ATAACTTCTC TCGATGACAC TTTCTTGGGT TTTGGTGGAT CCTTTGGGAA

12260 TTGCTGGAGC CTGGAGCATC TGAACTGTTT GTGGATTGAG AAGGCATTAC TTTAGACTGA

12320 CATGCAGCCA GTATTCTGTG AGATGCCCAG CCTAACAAAC CTAGATATCG GAGTTTATTT

12380 TCGATAAAAA TGGGTCTAGC TCTTTGCTTT CTTGCAGTTC CAACGGCACA TGTTTTATGA

12440 CAGTACTTGT AAGCAAGTTT CTTCCCCTGT ATATGGCGGT GAAGTCTGAT GGATACATTG

12500 TATGTTTTTC AGGCTTGCAT GATGTACCTG TTCATATTAA TTCAGATTGG CTTTGGAGAA

12560 AACCAAGTTT TCTTCGCAAG CTCAAGCAGT GGCCACTGCC TCACAGAAGA TCCTTCGCAC

12620 TGGGCGTGTC CATTTCCTCT TTGAACCATT GTTTGAGCAA CTGATTTTGT TCAATTCCAA

12680 TTTTTTGTAA TTTCTGCCCA GGATTCTTGT ATAACTGTAT GTAAAAGCAT TATAATGGAA

12740 ATTTGGATCA TAAAGTACGT TTGTTTCATA TGA

＞*LeEIN3*

-692 GTTTTTACTT TTAATGTCTT TGCACCTTCC TCTCTTCTTC TTACTTTCCA ATACACAACA

-632 AAAGTGAAAA CATCACTTCA ATACACCTAA CATTCTTCTC AAACCCCTTC TCTTTCTCTC

-572 TTTTTTCTTT TTTTTTGGTT CAAAATCAAA GTAGTATAGC CATAGATAAC AGCTTCAACA

-512 AGCTGTTTTC AAGAAAATCA TGTAAGTCAT TTTCTTGATT TTTTTTTGTT AAGTTTTTTT

-452 GAGTGTTGGT TTGTTGTGAA TTTTTGTGAA AATATGGTTC TTGAAGGGGT TGGTAGATCA

-392 GAGTATATGT GGGGATTAAA AGGGTGTTTT TTTTTTACAA ACAAAGAAGA TATAGATGGA

-332 ATTTTTAATA GTAATCAGTA GAGAAAATTA TGAAATTCTG TTAAAAGGGT GTTTTTCAGT

-272 AGAAAAAATT TGTTTTATGG CGTTGTTATA TTTATGTTTG TTTGTGTTTT TTTCTCCATG

-212 GAGTTATATT TATATGATGA CGTGTTATGT GTAGTATAAA GTGGGAGTAA TTTTTTATGA

-152 TTTGTTAAAG ATTATCTAGT TTTGAGGAAT TTGGACTGTT TTGTTTGATG ATGTTAGATT

-92 AAAGATCATT AATTTTGGAT TAAATTGGAG TTGATTTTAC TAAAAGTTGA GTTTATTTGT

-32 TGTATCTGAA TTGGTGTTGC AGAATTTGGT AA**ATG**GGGAT ATTTGAAGAT ATGGGGTTCT

29 CTGGAAATTT TGAGTTTCTA TCTGATTCTA TGGGATGTGG AGCTCAAGAA GTTGAGCATA

89 AGCCGGTTGG GTTGGAGGAG GATGATTATA GTGATGAGGA GATGGATGTG GAAGAGCTAG

149 AGAGGAGGAT GTGGAGGGAT CGAATGCTTT TGAGGCGTCT CAAAGAGAAA AACAAGAATA

209 AAGTGGTGGG GGATGGTGCG AAGCAGCGTC AGTCGCAGGA GCAGGCTCGT AGAAAGAAGA

269 TGTCGCGTGC ACAAGATGGT ATACTGAAGT ACATGCTGAA AATGATGGAG GTTTGTAATG

329 CTCAGGGTTT TGTTTATGGA ATTATCCCTG AGAAAGGGAA GCCTGTGACT GGTGCTTCGG

389 ACAATCTTCG TGCTTGGTGG AAGGAAAAGG TCAGATTTGA TCGAAATGGC CCTGCTGCTA

449 TTGCTAAGTA TCAGGCTGAT AATCAGATTC CTGGGAGAGT TGAGGAATCG AGTGTGATAG

509 TTTCCACTCC CCACACTTTA CAGGAGCTGC AGGATACAAC TCTAGGATCC CTTTTGTCTG

569 CTTTGATGCA GCACTGTGAT CCTCCACAGA GGCGGTTTCC GTTGGAGAAG GGGGTATCTC

629 CACCCTGGTG GCCCTCTGGT AAAGAGGAAT GGTGGGGTCA GTTGGGTCTG CCAAATGATC

689 AAGTTCAACC TCCATACAAG AAGCCTCATG ATCTGAAGAA GGCCTGGAAG GTTGGTGTTC

749 TGACGGCGGT AATCAAACAC ATCTCTCCCG ACATTGCTAA GATTCGCAAG CTTGTTCGAC

809 AGTCAAAGTG CTTGCAGGAT AAGATGACAG CTAAGGAGAG TGCTACTTGG CTTGCTATTA

869 TCAATCAAGA AGAGGCTTTG GCTCGTAAGC TGTATCCTGA CAGCTATCCA CAGGGATCTC

929 TAGCTGTTGG TAATGGTTCC TTTTTCATCA GCGATGCTAG CGATTACGAT GTGGAAGGAG

989 TGGATAACGA GAGAAACAAT GAAGTGGAAT GTAAACCCCA TGACATCAAT CTCCAAACTG

1049 GAATTATGTT ACCTAAAGAT AGGGTTTTGA TGCCAGGTTT AGCTCCAGTG AAAGGAGAAA

1109 TTATTGATTT AACTTCCGAT TTTATCCAGA AGAGGAAGGA ACCATGTTTT GAGGAGTCTG

1169 TTGATCAAAA GATATATACT TGTGAGTACC TTCACTGCCC ATACAGCAAT TATCAAGCTG

1129 GATTCCTTGA CAGGACTTCA AGAAACAACC ACCAAATGAG TTGTCCATTC CGGTTCAATT

1189 CTGCTCAAAC ACTTACTACA CCTAAGTATC AGATCAACTA TGAGCACAAC ACAGTTTTTC

1249 CTGCACAAAC TGCAACTTCT AAGCCAGCGG TCTCGTCAGT CACTGCTTCC TCTTCGATGA

1309 GTGCCTCGGG GCTTGGACTC CCTGAAGATG ATCAGAGGAT CATTTCTGAC CTCATAACAT

1369 CATACGACAA CAACTTTCAG CAAAATGGTA GCATCTGTTC CGGAATTTCT GAGATTCTAG

1429 TAAACCAAAG CCTGCCTCAG CAACAAACAG TTGAACTTCC CATGGATGGC AACATCAACC

1489 TAGGACATAT GGAGACCTCA GCTCAAGAAA CCAGCATGCC TGTTTATCGT TCAACAGAGT

1549 TTCAATATGA TCAATGCAAA ATGTCCTTTG ACGCCCCCTT CGGTGGAAAC ATAAATGATA

1609 TAACTGATTA CAGATTCGGT TCCCCGTTCA ACTTGGGAGG AAGCGACTAC GCCGTGGAAC

1669 AGCTGACAAA GCAGGATATA TCTACATGGT ACCTCTGAAC TAGTACTAGT ATTAAACTGT

1729 CTTATTTCCT ATATGAAGGC TTGATAGGTT GTATATGTTT AGATAAGTGA TCAACTCTCT

1789 GTCCTTTATA TATACAGGTA TTGTAGTTTC TTTGTGATGT AGTTGATGTT TCCATGGTTG

1849 TAAGTGCTAA ACACAATAAT TATCTTTATG CAATTATGTT TTAAACTCA

>SlERF-A1

-537 GATAACAATA AATGAAAGAA AAAACATATA CAATTATTCA AATTATCACC AAACCTATCC

-477 AACAACACAC TTCCACACCT CCCATAATAT TATCTCGATC AATCATATCA TAATTCTAAC

-417 GAAAAGCAAA AGAAAGACTT TAATGGAAGT CATGGTCCAT ATGTCTACGA CAAATAGTGA

-357 ATTACTAGAA AAATGCGGCT CAGTAATTTT ATCTTATTAG TATTATTCAA AATACCTAAT

-297 TTCCAATTTT CAAATACAAC ACGTGCTTAT TGAAACTCTC TATTTCCCTC TCAAAAACTT

-237 AGAACTTTTC TCCATTTAGA AGTTTCAAGG AAAGCTCAAA TTCCAAACAA ACAATATTTT

-177 AATCATTATT GTTATTAAAT TTTCCCACCC GTGCATGAAC CAAATTTCAT ATAAATATTT

-117 CCCTTTCCCC GCTTTAATCC AGGAAAAAAA AACTCATAAC TTCCTATTGT TTTTTCTTGA

-57 TTTGCTCTAT TTGTACTAAA TTCGCTTCGA TATAAAAATT CATAACCAAA ATTCAAA**ATG**

4 TATTCAAATT GTGAACTAGA AAATGATTTT TCAGTACTCG AATCAATTAG AAGATACTTA

64 CTTGAAGATT GGGAAGCTCC ATTAACGAGC TCTGAAAACT CAACATCCTC AGAGTTCAGC

124 CGGAGCAACA GCATTGAATC CAATATGTTT AGTAATTCAT TTGATTATAC ACCTGAAATT

184 TTTCAAAATG ATATTCTTAA TGAAGGATTT GGATTTGGAT TTGAATTCGA GACTTCTGAT

244 TTTATAATCC CTAAATTAGA GTCACAAATG TCAATCGAAT CACCTGAAAT GTGGAATTTA

304 CCGGAATTTG TGGCTCCATT AGAGACGGCG GCGGAGGTGA AAGTTGAAAC ACCGGTTGAG

364 ATGACAACTA CGACGACGAA GCCAAAGGCA AAGCATTATA GAGGTGTGAG AGTGAGGCCA

424 TGGGGGAAAT TCGCGGCGGA AATTAGAGAT CCGGCGAAAA ATGGAGCACG AGTTTGGCTC

484 GGTACATATG AGACGGCGGA GGATGCGGCG TTGGCTTACG ACAAGGCGGC TTTTCGCATG

544 CGGGGATCAC GTGCATTGCT GAATTTTCCG TTGAGGATTA ATTCCGGTGA ACCGGATCCT

604 GTTAGAGTTG GATCGAAGAG ATCGTCAATG TCGCCGGAGC ATTGTTCATC GGCGTCGTCG

664 ACGAAGAGGA GGAAGAAGGT TGCTCGTGGA ACAAAGCAAT AAGTCCTAAA AGTGGGCCCT

724 GTATAGTAAT AAAAAAAAAA TAGAATTATC CGACGGAAGT TGTTTTCTTA TAGAAAAAAG

784 TTATTATTTT GGGTGAAGTA ATGAATTTGT TTTTTCTTTC ATTTTTCGGC TGTGGTTGAT

844 GAAATCAACG AATCAAATAA TTATCCAAAA AATTAAGACA ATAATGTGAT AGTGATTATT

904 AAATAAATAA AAAGGTCAAA CTTACGTA

>LeERT10

-3 GTG**ATG**GTTA CCGGAGCTTC ATCGGGAATC GGTAGAGATT TCTGCTCCGA TTTATCAAAA

58 GCTGGTTGTA GAATCATCGC TGCCGCTCGT CGAATCGATC GATTGCAATC TCTATGTGAT

118 GAAATCAACT CGAATTCATC GAACGGATCG ACGAAGTCGA GTCAGGATTT ACGTGCCGTA

178 GCGATTGAGC TTGACGTTAG CGCTAATGGT TCTGCCATTG AAGCCGCCGT ACAGAAAGCT

238 TGGGATGCAT TTGGACGTAT CGACGGTTTG GTTAATAACG CCGGCTTTCG AGGTGAGATT

298 GGTTGATAAA TTATCGAATT GAATTCAGTA TTTTAGCGTA CCCAAGTATT AGCGATAGAT

358 TAGGAACGAA AAACAACCGA TAGTTGAACT TGAAATGACT AGTTATGAAT ATGAAACTCG

418 CGAAAAAGTG AGTGATGACC AATTGTGAAT ATGAAACTCG CGAAAAAGTG AGTGATGACC

478 AATTGTGAAT ATGAAACTCG
